# Mapping epidermal and dermal cellular senescence in human skin aging

**DOI:** 10.1111/acel.14358

**Published:** 2024-10-06

**Authors:** Grace T. Yu, Clarisse Ganier, David B. Allison, Tamara Tchkonia, Sundeep Khosla, James L. Kirkland, Magnus D. Lynch, Saranya P. Wyles

**Affiliations:** ^1^ Mayo Clinic Medical Scientist Training Program Mayo Clinic Graduate School of Biomedical Sciences, Mayo Clinic Alix School of Medicine Rochester Minnesota USA; ^2^ Centre for Gene Therapy and Regenerative Medicine King's College London, Guy's Hospital London UK; ^3^ Indiana University School of Public Health Bloomington Indiana USA; ^4^ Division of Endocrinology and Metabolism, Department of Medicine Center for Gerotherapeutics, Cedars‐Sinai Medical Center Los Angeles California USA; ^5^ Division of Endocrinology, Department of Medicine Mayo Clinic Rochester Minnesota USA; ^6^ Robert and Arlene Kogod Center on Aging Mayo Clinic Rochester Minnesota USA; ^7^ St. John's Institute of Dermatology King's College London, Guy's Hospital London UK; ^8^ Department of Dermatology Mayo Clinic Rochester Minnesota USA

**Keywords:** cellular senescence, dermis, epidermis, single‐cell gene expression analysis, skin aging, skin pathology, spatial analysis

## Abstract

Single‐cell RNA sequencing and spatial transcriptomics enable unprecedented insight into cellular and molecular pathways implicated in human skin aging and regeneration. Senescent cells are individual cells that are irreversibly cell cycle arrested and can accumulate across the human lifespan due to cell‐intrinsic and ‐extrinsic stressors. With an atlas of single‐cell RNA‐sequencing and spatial transcriptomics, epidermal and dermal senescence and its effects were investigated, with a focus on melanocytes and fibroblasts. Photoaging due to ultraviolet light exposure was associated with higher burdens of senescent cells, a sign of biological aging, compared to chronological aging. A skin‐specific cellular senescence gene set, termed SenSkin™, was curated and confirmed to be elevated in the context of photoaging, chronological aging, and non‐replicating *CDKN1A*+ (p21) cells. In the epidermis, senescent melanocytes were associated with elevated melanin synthesis, suggesting haphazard pigmentation, while in the dermis, senescent reticular dermal fibroblasts were associated with decreased collagen and elastic fiber synthesis. Spatial analysis revealed the tendency for senescent cells to cluster, particularly in photoaged skin. This work proposes a strategy for characterizing age‐related skin dysfunction through the lens of cellular senescence and suggests a role for senescent epidermal cells (i.e., melanocytes) and senescent dermal cells (i.e., reticular dermal fibroblasts) in age‐related skin sequelae.

## Graphical abstract

Bioinformatic analysis of scRNA‐seq and spatial transcriptomics of human skin aging revealed increased senescent cells, identified as *CDKN1A*+ non‐replicating cells, with sun exposure and chronological age. Senescent melanocytes in the epidermis expressed increased melanin biosynthesis, while senescent fibroblasts in the reticular dermis expressed decreased collagen and elastic fiber genes. Senescent cells showed a tendency to cluster, and their phenotypes were inferred to change with time. The graphical abstract figure was created with BioRender.com.

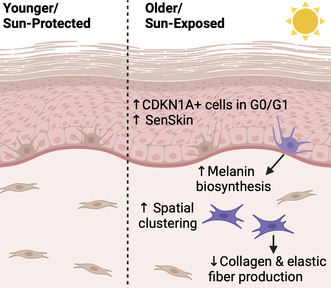

**Abbreviations**
E: early
ECM: extracellular matrix
L1: late 1
L2: late 2
miRNA: microRNA
NIH: National Institutes of Health
OCT: optimal cutting temperature
PCA: principal component analysis
QQ plot: quantile‐quantile plot
RNA: ribonucleic acid
SASP: senescence‐associated secretory phenotype
scRNA‐seq: single‐cell RNA sequencing
UV: ultraviolet

## 1 INTRODUCTION

Advances in single‐cell RNA sequencing (scRNA‐seq) and spatial transcriptomic platforms are revolutionizing our understanding of skin health across lifespan (Almet et al., 2023; Farlik & Weninger, 2023). Multiscale skin atlases, such as the developing Human Skin Cell Atlas, provide opportunities to uncover novel cell types, cell lineages, and their spatial localization (Almet et al., 2023; Ganier et al., 2024). These atlases facilitate deep investigation of skin aging, across a wide range of chronological ages and skin dermatomes, including sun‐exposed and sun‐protected areas, at single‐cell resolution to identify senescent cells.

'Beyond chronological aging, biological aging is a functional definition that describes the loss of physiological integrity that progresses with chronological age and accelerates with stressors, such as ultraviolet (UV) light exposure (López‐Otín et al., 2013, 2023). scRNA‐seq and spatial technologies can measure the hallmarks of biological aging, including cellular senescence, altered intercellular communication, loss of proteostasis, and chronic inflammation. Notably, single‐cell resolution is crucial in identifying senescent cells and associated transcriptional alterations.

Both intrinsic and extrinsic stressors can induce cellular senescence as a defense mechanism, which is characterized by irreversible cell cycle arrest, apoptotic resistance, and the senescence‐associated secretory phenotype (SASP). Heterogeneity in senescent cell phenotypes has impeded the identification of sensitive and specific biomarkers, resulting in new initiatives, such as the National Institutes of Health (NIH) SenNet Consortium, to identify high‐resolution and cell‐specific characterization of cellular senescence as relevant to human lifespan (Lee et al., 2022). Moreover, scRNA‐seq facilitates simultaneous analysis of the expression of tens of thousands of genes that could altogether provide a coherent identification of skin senescence‐associated features. Furthermore, spatial transcriptomics can provide spatial maps of senescent cells, including compartmentalization to specific tissue locations or patterns of spatial behaviors, such as clustering or dispersing.

Herein, we mapped epidermal and dermal senescent cells using scRNA‐seq and spatial transcriptomic human skin atlases (Ganier et al., 2024; Solé‐Boldo et al., 2020; Tabib et al., 2018). In this quest, we identified novel combinations of differentially expressed genes and gene sets to define cellular senescence in skin, induced by chronological aging and photoaging, in epidermal melanocytes and dermal fibroblasts. Using these definitions of senescent skin cells, we discerned the effects of cellular senescence on epidermal and dermal functions of human skin, including skin pigmentation and extracellular matrix (ECM) synthesis. Moreover, we elucidated changes in gene expression underlying early and late senescent phenotypes. Finally, we evaluated the spatial localization of senescent skin cells. To our knowledge, this is the first study to integrate scRNA‐seq with spatial transcriptomic profiling for the characterization of senescent cells in human adult skin.

## 2 RESULTS

### 2.1 *CDKN1A* (p21) versus *CDKN2A* (p16) as cellular senescence markers in human adult skin

**FIGURE 1:**
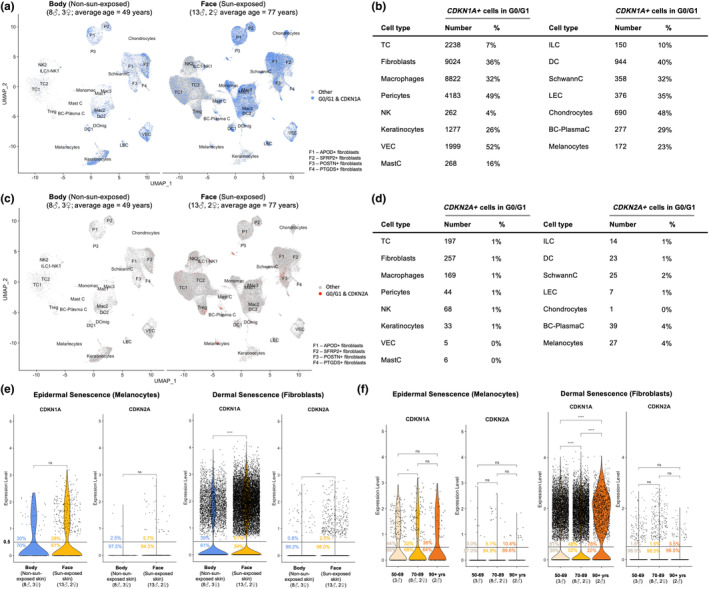
*CDKN1A* (p21) versus *CDKN2A* (p16) as cellular senescence markers. (a, b) *CDKN1A*+ cells in G0/G1 of cell cycle. (c, d) *CDKN2A*+ cells in G0/G1 of cell cycle. (e) *CDKN1A* and *CDKN2A* expression versus anatomic location and sun exposure in fibroblasts and melanocytes. (f) *CDKN1A* and *CDKN2A* expression versus age in fibroblasts and melanocytes in sun‐exposed skin. ns: *p* > 0.05; *p ≤ 0.05; **p ≤ 0.01; ***p ≤ 0.001; ****p ≤ 0.0001.

Three scRNA‐seq datasets of human skin were integrated, and their batch effects were corrected, amounting to a total of 116,627 cells of 15 cell types and 27 subtypes (Figure S1a,b) (Ganier et al., 2024;Solé‐Boldo et al., 2020; Tabib et al., 2018). One dataset contained samples of skin from the face (Ganier et al., 2024) where there is greater sun exposure, while the other two datasets contained samples of skin from the body (Solé‐Boldo et al., 2020; Tabib et al., 2018), which has less to minimal sun exposure. Chronological age could confound these results as the average age of the face samples was higher than the body samples, but like sun exposure, chronological age is also associated with increased senescent cell burden. Well‐established transcriptomic markers for cellular senescence include the cell cycle inhibitors, *CDKN1A*, which produces the protein p21, and *CDKN2A*, which yields p16. Moreover, senescent cells are, by definition, non‐replicating (G0/G1 phases). Hence, we evaluated *CDKN1A* and *CDKN2A* expression of non‐replicating cells in the scRNA‐seq skin datasets after cell cycle analysis (Figures 1a,c and S1c). Both *CDKN1A+* and *CDKN2A*+ non‐replicating cells increased with greater sun exposure in skin of the face versus the body. Increases in *CDKN1A*+ non‐replicating cells were also notable in fibroblasts, pericytes, macrophages, and dendritic cells (Figure 1a), while increases in *CDKN2A*+ non‐replicating cells were notable in fibroblasts, melanocytes, and B cells/plasma cells (Figure 1c). *CDKN1A*+ non‐replicating cells comprised 4%–52% of skin cell types (Figure 1b). Substantially fewer *CDKN2A*+ non‐replicating cells were observed; *CDKN2A*+ non‐replicating cells ranged from 0% to 4% of skin cell types (Figure 1d). Hundreds to thousands of non‐replicating cells of each cell type expressed *CDKN1A*, providing robust numbers for analysis, while *CDKN2A* expression was only observed in 1 to 257 cells of each cell type, limiting the power to analyze *CDKN2A*+ cells in these datasets.

In the epidermis and dermis, senescent cell types that drive human skin aging are primarily melanocytes and fibroblasts, respectively (Victorelli et al., 2019; Wlaschek et al., 2021; Wyles et al., 2023). Hence, *CDKN1A* and *CDKN2A* expression was compared in the context of photoaging and chronological aging. Both *CDKN1A* and *CDKN2A* increased significantly with greater sun exposure in fibroblasts located in the face versus the body (Figure 1e). We did not observe compelling evidence to support changes in *CDKN1A* and *CDKN2A* expression with sun exposure in melanocytes. Changes in *CDKN1A* and *CDKN2A* expression with chronological aging in sun‐exposed skin were mixed; *CDKN1A* decreased in melanocytes from the 70 to 89‐year‐old category compared to the 50 to 69‐year‐old category, and, in fibroblasts, it decreased then increased going from the 50 to 69‐year‐old to the 70 to 89‐year‐old to the 90+ year‐old categories (Figure 1f). However, as damage from sun exposure could accumulate with age, analyses of chronological aging could be confounded by photoaging.

### 2.2 A novel panel of skin senescence markers, SenSkin™

Although *CDKN1A* is a marker of cellular senescence, further analysis with a set of genes could improve sensitivity and specificity of senescent cell identification. A new panel of genes associated with cellular senescence in skin, termed SenSkin™, was curated by two domain experts who reviewed peer‐reviewed papers (Table S1) (Ahlers et al., 2021; Solé‐Boldo et al., 2020; Zou et al., 2021). The SenSkin™ collection includes genes related to apoptosis, ferroptosis, metabolism, cell migration, and the SASP, including pro‐inflammatory and ECM‐related genes. In total, 166 genes were identified in the literature, of which 165 were detected on scRNA‐seq; 164 genes were upregulated and one, *LMNB1*, was downregulated in senescent cells.

**FIGURE 2:**
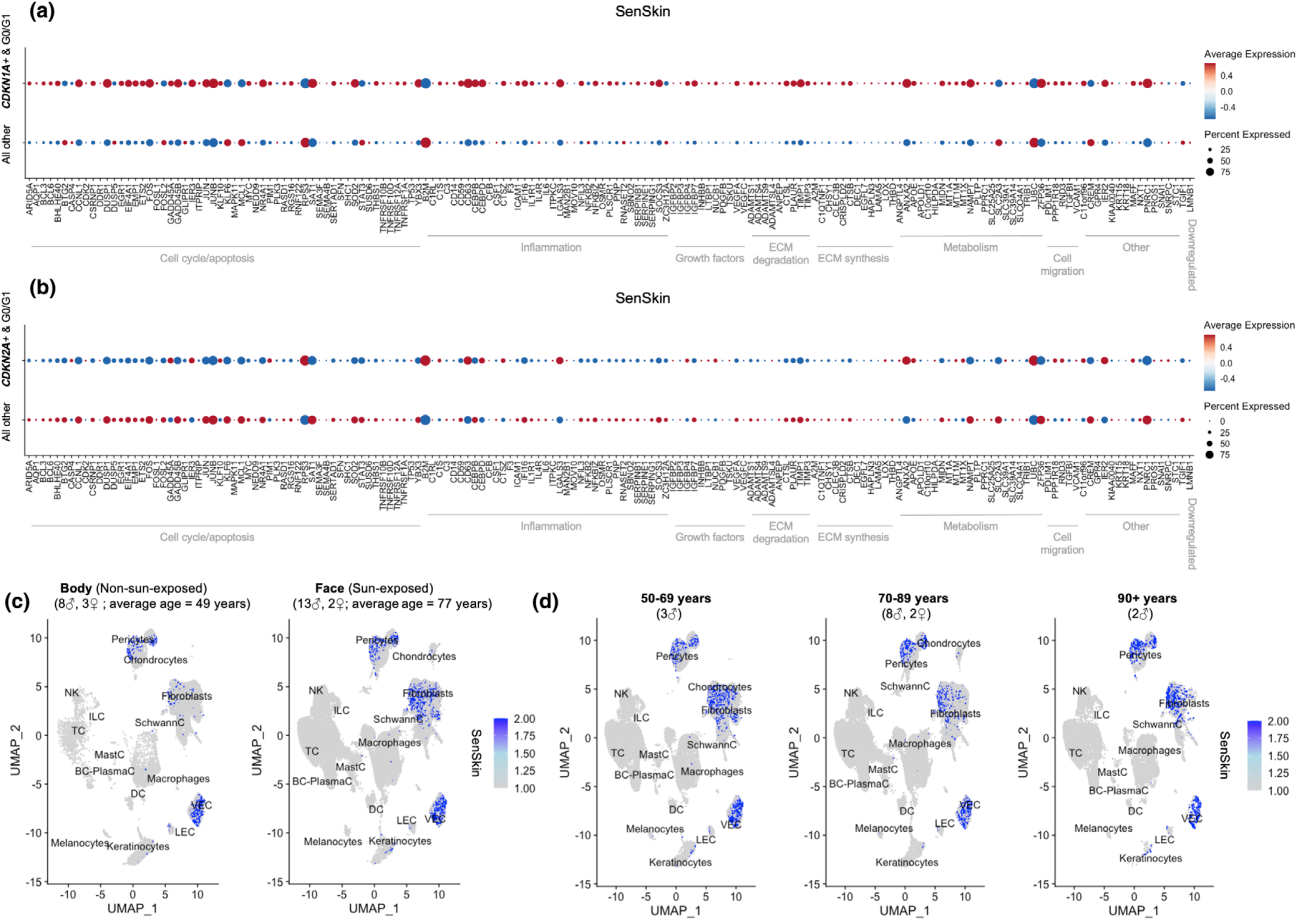
A novel panel of skin senescence markers, SenSkin™. SenSkin™, were grouped approximately by gene function based on review of GeneCards (Stelzer et al., 2016), in (a) *CDKN1A*+ cells in G0/G1 (139/165 genes congruent) and (b) *CDKN2A*+ cells in G0/G1 (44/165 genes congruent) versus all other cells. SenSkin™ was more congruent with *CDKN1A*+ cells in G0/G1 than *CDKN2A*+ cells in G0/G1 (*p* < 2.2 × 10^−16^). Red dots represent gene upregulation, and blue dots represent gene downregulation. Dot size represents percent of cells expressing the gene. (c) SenSkin™ composite score versus sun exposure. (d) SenSkin™ composite score in sun‐exposed skin versus age.

Gene expression of SenSkin™ was compared in *CDKN1A*+ non‐replicating cells versus all other cells (i.e., *CDKN1A*‐ cells or replicating cells) and *CDKN2A*+ non‐replicating cells versus all other cells (i.e., *CDKN2A*‐ cells or replicating cells) (Figure 2a,b). Specifically, congruence was evaluated as the sum of the number of genes upregulated in senescent skin cells that were upregulated in the subpopulation of interest and number of genes downregulated in senescent skin cells, namely *LMNB1*, that were downregulated in the subpopulation of interest. The SenSkin™ expression profile had 139/165 = 84% genes congruent with *CDKN1A*+ non‐replicating cells and only 44/165 = 27% genes congruent with *CDKN2A*+ non‐replicating cells, suggesting greater congruence with *CDKN1A*+ non‐replicating cells than *CDKN2A*+ non‐replicating cells (*p* < 2.2 × 10^−16^). When evaluating specific cell types, SenSkin™ gene expression was more congruent with *CDKN1A*+ non‐replicating melanocytes (64%) than *CDKN2A*+ non‐replicating melanocytes (39%) (*p* = 1.0 × 10^−10^) (Figure S2a,b). SenSkin™ genes upregulated in *CDKN1A*+ non‐replicating melanocytes were related to wound healing, coagulation, and apoptosis pathways (Figure S2e). Similarly, SenSkin™ was more congruent with *CDKN1A*+ non‐replicating fibroblasts (73%) than *CDKN2A*+ non‐replicating fibroblasts (47%) (*p* = 2.8 × 10^−11^) (Figure S2c,d). In *CDKN1A*+ non‐replicating fibroblasts, upregulated SenSkin™ genes were related to alpha‐beta T cell activation, apoptosis, and mononuclear cell differentiation pathways (Figure S2f).

Because cellular senescence phenotypes are thought to be tissue‐specific, the same analysis was performed with genes from the SenMayo gene set (Saul et al., 2022), an established panel that was tested on bone marrow and adipose tissue in humans and mice, but not on skin. Likewise, SenMayo was more congruent with *CDKN1A*+ non‐replicating cells (74%) than *CDKN2A*+ non‐replicating cells (40%) (*p* = 6.2 × 10^−15^) (Figure S3a,b). However, SenSkin™ genes (84%) displayed more congruence with *CDKN1A*+ non‐replicating cells than SenMayo genes (74%) in this human skin dataset (*p* = 0.0017).

Gene expression in SenSkin™ was then evaluated as a composite score, specifically, a normalized sum of *z*‐scores of upregulated genes subtracted by the downregulated gene. In evaluating SenSkin™ across all cell types, fibroblasts, vascular endothelial cells, and pericytes displayed the highest SenSkin™ composite scores (Figures 2c and S2g). As expected, the SenSkin™ composite score increased with sun exposure, particularly in keratinocytes and fibroblasts (Figures S5c and S6a). No consistent increases or decreases in the SenSkin™ composite score were observed in facial skin across the three age groups, but it increased in keratinocytes, fibroblasts, pericytes, and vascular endothelial cells in the above‐90‐year‐old group (Figures 2d, S2h, S5d and S6b). In contrast, the SenMayo composite score was higher in fibroblasts, vascular endothelial cells, chondrocytes, and macrophages (Figure S3c). Chronological aging was not observed to have consistent increases or decreases in SenMayo composite scores across the three age groups, but photoaging was associated with higher SenMayo in fibroblasts, macrophages, and vascular endothelial cells (Figure S3d,e). Hence, the SenSkin™ gene set could serve as a valuable tool for investigating cellular senescence specific to human skin.

### 2.3 Senescent melanocytes are associated with features of epidermal aging

**FIGURE 3:**
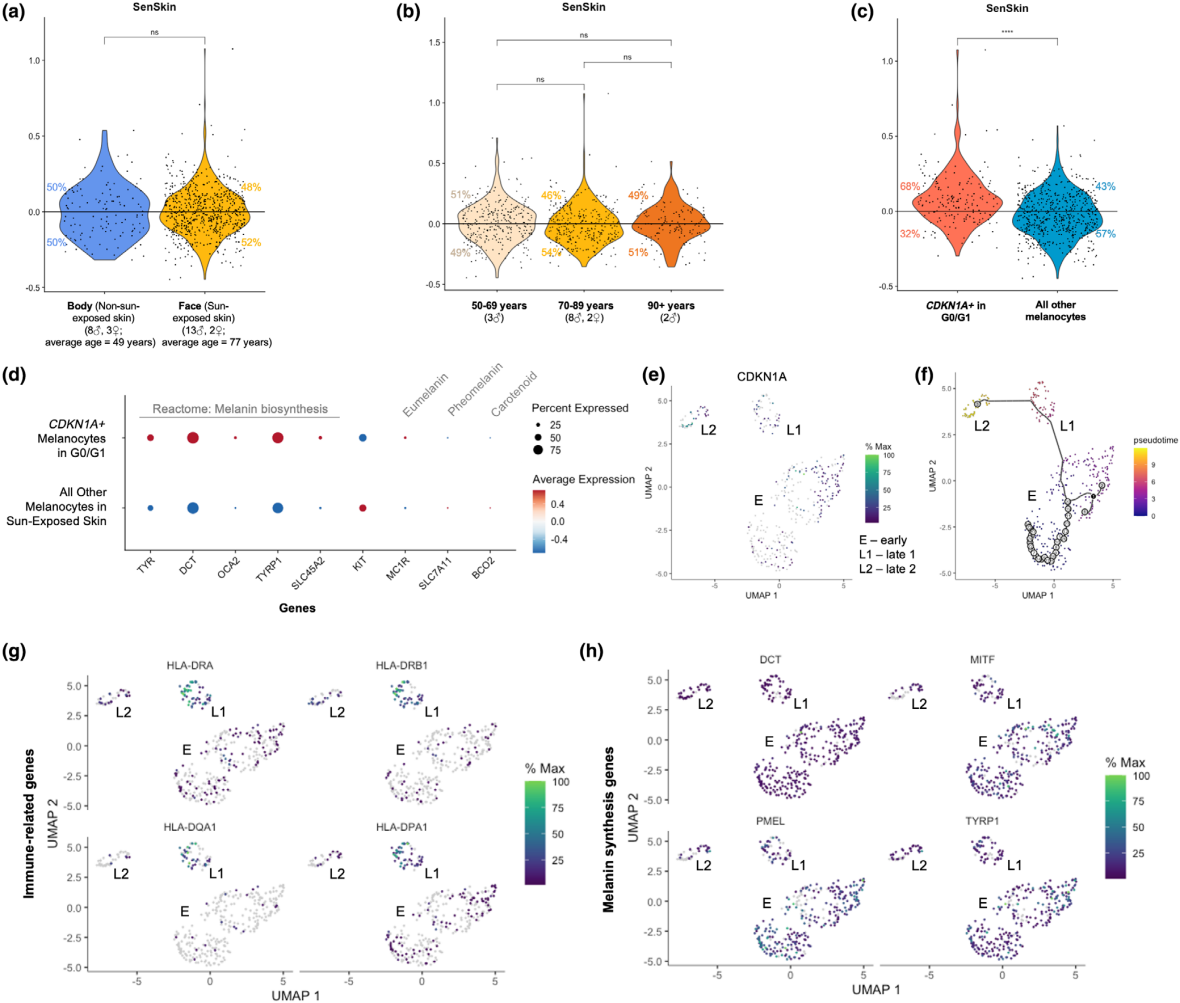
Epidermal senescence is driven by *CDKN1A*+ non‐replicating melanocytes in human adult skin. SenSkin™ composite score in (a) non‐sun‐exposed versus sun‐exposed melanocytes, (b) sun‐exposed melanocytes with age, and (c), non‐replicating *CDKN1A*+ versus all other melanocytes. (d) Melanin synthesis gene expression in non‐replicating *CDKN1A*+ melanocytes versus all other melanocytes in sun‐exposed skin; specifically, the Reactome melanin biosynthesis gene set and genes for c‐kit (*KIT*), eumelanin (*MC1R*), pheomelanin (*SLC7A11*), and carotenoid metabolism (*BCO2*). Red dots represent gene upregulation, and blue dots represent gene downregulation. Dot size represents percent of cells expressing the gene. (e) *CDKN1A* expression across non‐replicating melanocyte clusters from sun‐exposed skin, labelled clusters E (early), L1 (late 1), and L2 (late 2). (f) Pseudotime across clusters. Higher pseudotimes represents later timepoints in cell trajectories. (g) Immune‐related genes across non‐replicating melanocyte clusters. (h) Melanin synthesis genes across non‐replicating melanocyte clusters. ns: *p* > 0.05; *p ≤ 0.05; **p ≤ 0.01; ***p ≤ 0.001; ****p ≤ 0.0001.

Senescent melanocytes have been described as the primary driver of epidermal aging (Victorelli et al., 2019; Waaijer et al., 2015; Wyles et al., 2023). First, the SenSkin™ composite score in melanocytes was evaluated in the context of photoaging and chronological aging. The dataset did not reveal compelling evidence supporting changes in the SenSkin™ composite score in the context of photoaging or chronological aging of sun‐exposed skin (Figure 3a,b). However, SenSkin™ composite score was significantly higher in non‐replicating *CDKN1A*+ melanocytes compared to all other melanocytes, suggesting that SenSkin™ is associated with a subset of melanocytes, such as senescent melanocytes, rather than global changes due to aging (Figure 3c).

The primary role of melanocytes is to produce melanin to protect the skin from UV damage. Hence, to evaluate the functional effect of cellular senescence in melanocytes, melanin biosynthesis‐associated gene expression was compared. Melanocyte populations from facial skin and body skin clustered separately; as such, analysis was performed on melanocytes from facial skin, a context in which melanin synthesis is stimulated. *CDKN1A*+ non‐replicating melanocytes had upregulation of all genes in Reactome's melanin biosynthesis gene set (Jupe, 2015) (Figure 3d). Differentiating by pigment type, they had upregulation of eumelanin‐related gene *MC1R* (Nasti & Timares, 2015), but they had downregulation of the pheomelanin‐related gene, *SLC7A11* (Chintala et al., 2005), and carotenoid‐related gene, *BCO2* (Suzuki & Tomita, 2022). Eumelanin is a brown to black pigment that is highly effective in dissipating UV radiation. Hence, increased eumelanin and melanin production could be a result of cellular senescence or a melanocytic response to UV exposure (Nasti & Timares, 2015). *KIT*, a melanocyte proliferation and survival gene, was downregulated in *CDKN1A*+ non‐replicating melanocytes, further corroborating the possibility that these melanocytes are non‐replicating and senescent (Wehrle‐Haller, 2003).

To make preliminary inferences about cellular senescence as a melanocyte cell fate, pseudotime analysis was performed on all non‐replicating melanocytes from sun‐exposed skin. Pseudotime describes methods of trajectory inference that orders cells based on dimension reduction or inferring topology (Saelens et al., 2019). The method we used in this analysis, Monocle, is based on independent component analysis reduction to one dimension, which allows for inference of linear or tree trajectories but does not allow for the inference of cycles or disconnected trajectories (Trapnell et al., 2014). Since melanocytes from sun‐protected skin clustered separately from melanocytes from sun‐exposed skin; as, sun‐exposed melanocytes were analyzed because UV exposure is expected to induce cellular senescence. After clustering melanocytes (Figure S4a), low *CDKN1A* expression and low SenSkin™ composite scores were used to infer the origin, or root node, of pseudotime (Figures 3e and S4b). Trajectory inference revealed a linear relationship between three clusters, from early (E) to late 1 (L1) to late 2 (L2) (Figures 3f and S4c). Cluster L2 (late 2) had higher *CDKN1A* than E (early), and *CDKN2A* and SenSkin™ appeared to increase with pseudotime, suggesting that they could represent groups of early and late senescent melanocytes respectively (Figure S4d). As pseudotime progressed, *CDKN1A* and *CDKN2A* decreased slightly before increasing, consistent with the time it takes cellular senescence to develop following cellular damage (Figure S4e).

Differential expression analysis was performed on the clusters, revealing significant differences in immune and melanin synthesis‐related genes (Figure 3g,h). Specifically, HLA genes appeared upregulated in L1 (late 1), suggesting that early senescent cells attempted to attract immune cells for clearance before expression was dampened in L2 (late 2). Melanin synthesis genes were primarily upregulated in E (early), suggesting that melanin synthesis decreased in later phases of senescence.

Emerging evidence suggests that keratinocytes could become senescent and contribute to skin aging as well (Contrepois et al., 2017; Rübe et al., 2021). Expression of *CDKN1A* and the SenSkin™ composite score increased with sun exposure but decreased with chronological age, while *CDKN2A* expression did not change significantly (Figure S5a–d). Like melanocytes, *CDKN1A*+ non‐replicating keratinocytes had a higher SenSkin™ composite score than other keratinocytes (Figure S5e). SenSkin™ genes had 48% congruence with *CDKN1A*+ non‐replicating keratinocytes and 45% congruence with *CDKN2A*+ non‐replicating keratinocytes, in contrast to 84% congruence with melanocytes (Figure S5f,g).

### 2.4 Senescent fibroblasts are associated with features of reticular dermal aging

**FIGURE 4:**
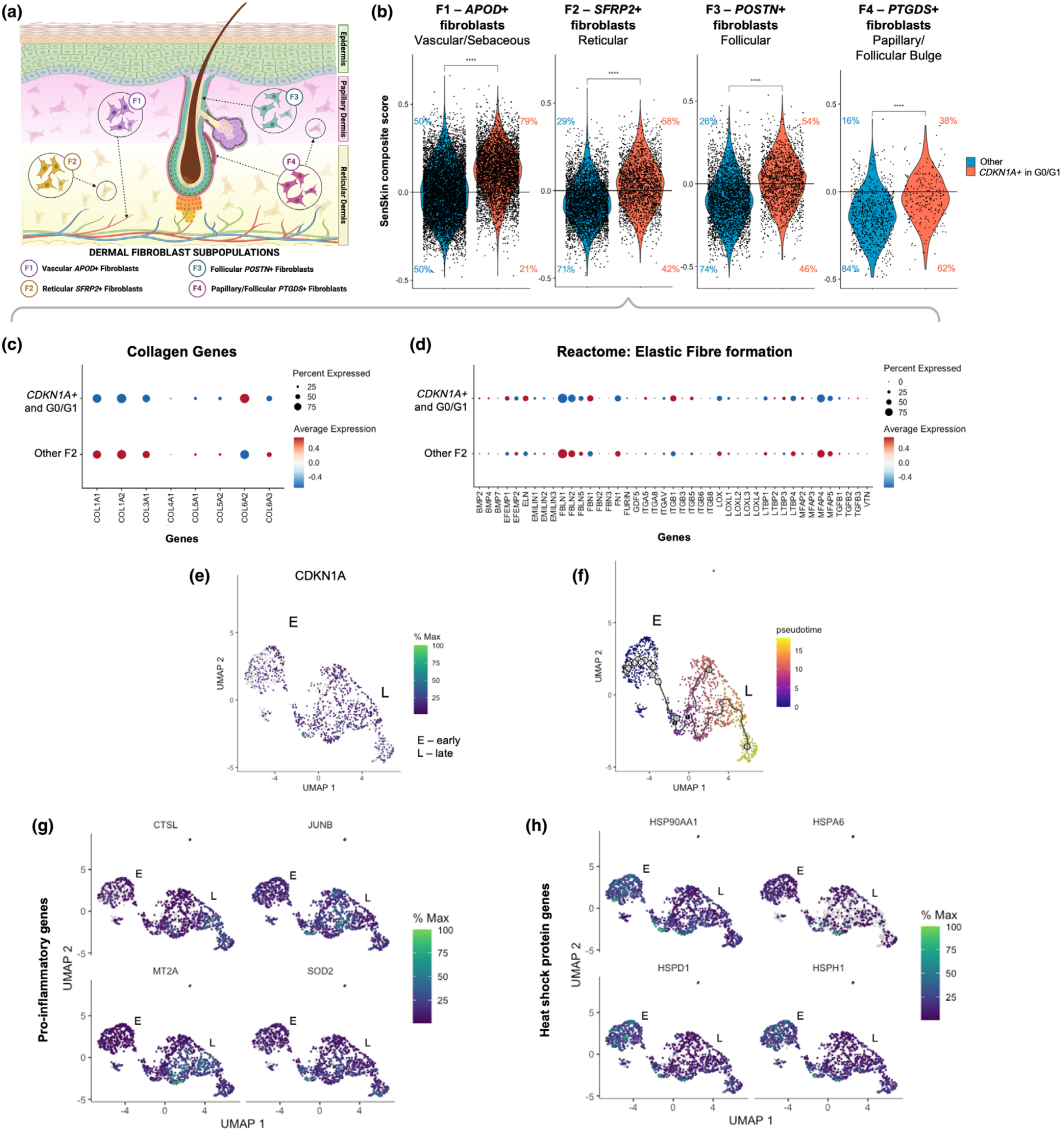
Dermal senescence is driven by *CDKN1A*+ non‐replicating reticular fibroblasts in human adult skin. (a) Localization of fibroblast subpopulations. Created with BioRender.com. (b) SenSkin™ composite score across fibroblast subpopulations in non‐replicating *CDKN1A*+ fibroblasts versus other fibroblasts in the same subpopulation. (c) Collagen‐related genes and (d) elastic fiber‐related genes given by Reactome Elastic Fibre Formation gene set (except BMP10, which did not meet scRNA‐seq quality control) in *CDKN1A*+ non‐replicating reticular dermal (F2) fibroblasts versus other reticular dermal fibroblasts. Red dots represent gene upregulation, and blue dots represent gene downregulation. Dot size represents percent of cells expressing the gene. (e) *CDKN1A* expression across clusters of non‐replicating F2 fibroblasts from sun‐exposed skin, labelled clusters E (early) and L (late). (f) Pseudotime across clusters. Higher pseudotimes represent later timepoints in cell trajectories. (g) Proinflammatory gene expression across clusters. (h) Heat shock protein genes across clusters. ns: *p* > 0.05; *p ≤ 0.05; **p ≤ 0.01; ***p ≤ 0.001; ****p ≤ 0.0001.

Fibroblasts are the most abundant cell type in the dermis, and they are responsible for dermal structural integrity, which declines with age and UV exposure and results in the clinical appearance of aged skin. Human dermal fibroblasts can be grouped into four subpopulations by their spatial localization, as previously described: F1 *APOD*+ fibroblasts associated with vasculature, F2 *SFRP2*+ fibroblasts associated with the reticular dermis, F3 *POSTN*+ fibroblasts associated with hair follicles, and F4 *PTGDS*+ fibroblasts associated with the papillary dermis and hair follicles (Figure 4a) (Ganier et al., 2024). All fibroblast subpopulations had higher SenSkin™ composite scores in the *CDKN1A*+ non‐replicating group (Figure 4b). Overall, the SenSkin™ composite score of fibroblasts was elevated with sun exposure but mixed with chronological age (Figure S6a,b).

Reticular dermal (F2 *SFRP2*+) fibroblasts maintain the ECM proteins of the dermis, an important structural component of skin. Hence, changes to reticular dermal fibroblasts with cellular senescence were investigated. The primary role of reticular dermal fibroblasts is ECM maintenance. Hence, the effects of cellular senescence on the synthesis of two major ECM components, collagen and elastic fibers, were elucidated. *CDKN1A*+ non‐replicating reticular dermal fibroblasts had downregulation of all the major collagen genes except *COL6A2* (Figure 4c) (McGrath et al., 2012). Similarly, *CDKN1A*+ non‐replicating reticular dermal fibroblasts had downregulation of the majority of genes in Reactome's elastic fiber formation gene set (Figure 4d) (Jupe, 2012). These trends were not observed in other fibroblast subpopulations with this dataset; vascular F1 *APOD*+ fibroblasts and papillary/follicular F4 *PTGDS*+ fibroblasts showed upregulation and downregulation of different collagen and elastic fiber‐related genes with *CDKN1A* expression, and follicular F3 *POSTN*+ fibroblasts displayed upregulation of collagen and elastic fiber‐related genes with *CDKN1A* expression, consistent with previous observations of different senescent phenotypes depending on cell types and lineages (Figure S6c–h).

For preliminary investigation into UV‐induced cellular senescence, pseudotime analysis was performed on reticular dermal fibroblasts from sun‐exposed skin, as they clustered separately from reticular dermal fibroblasts from sun‐protected skin. After clustering the reticular dermal fibroblasts from facial skin (Figure S7a), the cluster of reticular dermal fibroblasts with the lowest *CDKN1A* and *CDKN2A* expression as well as SenSkin™ composite scores was designated the origin for pseudotime analysis (Figure 4e and S7b,d). Of the two connected clusters, because one cluster had lower *CDKN1A* than the other, they were labeled as early (E) and late (L) respectively (Figure 4f and S7c). Progression of pseudotime was associated with an increase then decrease of *CDKN1A* and *CDKN2A* expression (Figure S7e). Differential expression analysis of clusters E (early) and L (late) revealed significant differences in pro‐inflammatory and heat shock protein genes (Figure 4g,h). Pro‐inflammatory gene expression was upregulated in cluster L (late), suggesting development of a SASP. Expression of heat shock proteins decreased in cluster L (late), suggesting a loss of proteostasis, which is a distinct hallmark of aging (López‐Otín et al., 2013; López‐Otín et al., 2023).

### 2.5 Spatial transcriptomics localization of cellular senescence markers in human adult skin

**FIGURE 5:**
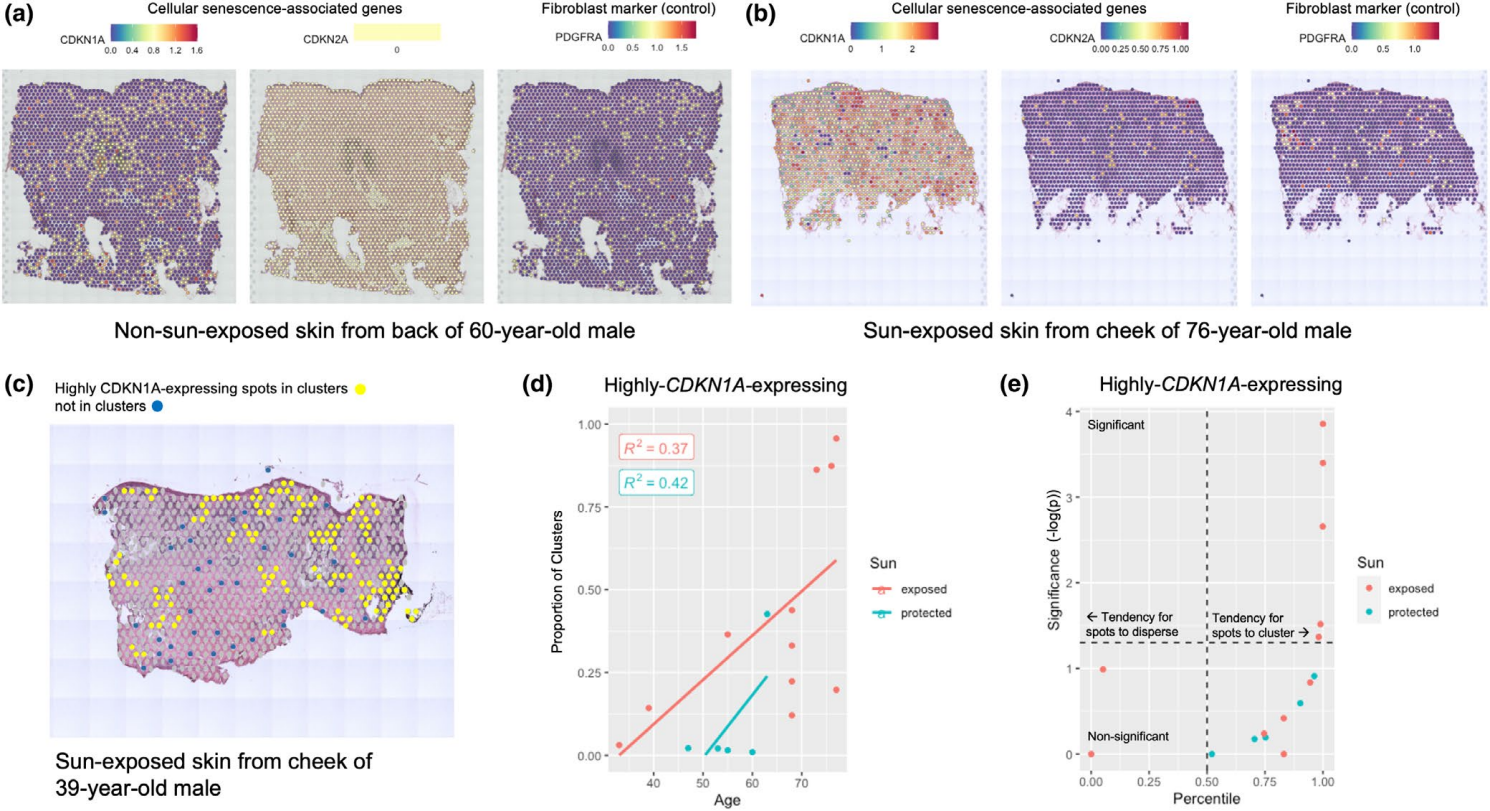
Spatial transcriptomics localization of cellular senescence markers in human adult skin. *CDKN1A* (p21) and *CDKN2A* (p16) spatial localization compared to *SDHA* housekeeping gene in (a) body skin (less sun‐exposed) and (b) facial (more sun‐exposed) skin. The color at each grid location corresponds to the magnitude of expression for each marker in the tissue section (blue representing none or low expression, yellow, medium expression, and red, high expression). (c) Example of clustering, with colored spots signifying high *CDKN1A* expression. Yellow spots represent spots that are in a cluster, that is, spots that have positive adjacent dots, and blue dots represent dots that are not in clusters, that is, have no positive adjacent dots. (d) Proportion of spots in clusters of highly‐*CDKN1A*‐expressing spots. (e) Tendency for clustering of highly‐*CDKN1A*‐expressing spots, where percentile >0.5 indicates a tendency for spots to cluster, and percentile <0.5 indicate a tendency for spots to disperse. *Samples removed if not express gene at threshold (SCT‐transformed gene expression values of 1 for *CDKN1A*‐highly‐expressing).

Spatial transcriptomic (Visium platform) datasets displayed spatial patterns of *CDKN1A* and *CDKN2A* expression in sun‐exposed and non‐sun‐exposed human skin (Figure 5a,b). Emerging studies have introduced the concept that senescent cells often exist in spatial clusters (Gurkar et al., 2023; Lee et al., 2018). To define clusters of senescent cells in Visium sections, any positive spots that were directly adjacent to each other, as computed using point‐to‐point distances, were designated as forming a cluster (Figure 5c).

First, the proportion of highly‐*CDKN1A*‐expressing spots that were in clusters increased with sun exposure and had a weak association with chronological age (Figure 5d). In contrast, a weaker trend was observed in *CDKN1A*+ spots, and no trends were observed in *CDKN2A*+ spots (Figure S8a,b). As a control, the housekeeping gene *SDHA* and fibroblast marker *PDGFRA* did not trend with age or sun exposure (Figure S8c,d). Moreover, the tendency for spots to cluster or disperse was evaluated using statistical simulations. To investigate clustering in both sun‐exposed and non‐sun‐exposed skin, highly‐*CDKN1A*‐expressing spots were defined with a normalized threshold of 1.0, which translated to the top 15% of *CDKN1A* expression in sun‐protected skin of the body and the top 46% of *CDKN1A* expression in sun‐exposed skin of the face. Higher thresholds excluded most non‐sun‐exposed samples from the body because they had lower *CDKN1A* expression overall. Highly‐*CDKN1A*‐expressing spots in sun‐exposed human skin samples clustered significantly more than expected given random chance, with five out of eleven of the sun‐exposed samples clustering significantly (Figure 5e). No significant tendencies for highly‐*CDKN1A*‐expressing spots to cluster or disperse were observed in non‐sun‐exposed samples. A similar finding was observed in *CDKN2A*+ spots, but two sun‐exposed samples in *CDKN1A*+ spots dispersed significantly more than expected, while four non‐sun‐exposed samples clustered significantly more than expected (Figure S8e,f). In comparison, the housekeeping gene *SDHA* clustered significantly more than expected in only two of eleven sun‐exposed samples (Figure S8g), and no tendencies for the fibroblast marker *PDGFRA* to cluster or disperse were observed in any samples (Figure S8h).

## 3 DISCUSSION

In human adult skin, a distinct profile of senescent cells contributes to epidermal vs. dermal aging. Senescent melanocytes in the epidermis and senescent fibroblasts in the dermis exhibit variable expression depending on inducers of senescence, that is, chronological aging versus photoaging. We describe a method to identify senescent cells in scRNA‐seq and spatial transcriptomic data, by combining high *CDKN1A* expression and cell cycle arrest, which can be applied to study normal and diseased human skin. We also developed SenSkin™, a novel gene set of cellular senescence markers in human skin, which could be used in analyses to further investigate the contribution of senescent skin cells in aging and age‐related diseases.

Current efforts are focused on expanding scRNA‐seq and spatial transcriptomic datasets in skin, that is, the Human Skin Cell Atlas (Almet et al., 2023), and for cellular senescence research, that is, NIH SenNet Consortium (Lee et al., 2022). This work exemplifies analyses that could be conducted with these databases. However, the datasets used in this work had a total of 26 samples, limiting the range of age, sex, skin locations, and Fitzpatrick skin types. Moreover, facial skin was assumed to have more sun or UV exposure than skin from locations on the body; however, skin from different parts of the face or body have varying amounts of sun exposure. Additional samples will be crucial for understanding differences due to these factors. However, the statistical methodology ensured robustness of the observed results. The limited sample size and use of Bonferroni corrections increased the chances of type II error, that is, that existent effects were not detected because of limited power. On the other hand, the chance that non‐existent effects were observed as significant, or type I error, was controlled by setting the significance level to 0.05 and using Bonferroni corrections. Wilcoxon signed‐rank tests used are also relatively robust to assumption violations and did not rely on asymptotic properties.

Biological aging accounts for intrinsic factors, such as chronological aging, and extrinsic factors, such as photoaging. This study suggested that photoaging plays a larger role than chronological aging in senescent cell accumulation, consistent with a study on photoaging and chronological aging in skin miRNAs (Srivastava et al., 2018). It has also been proposed that different methods or pathways of senescence induction yield variable senescent cell phenotypes. Here, strong associations were observed with sun exposure, high *CDKN1A* expression, and SenSkin™, which included SASP genes, suggesting that sun exposure, like other damage stimuli such as fracture (Chandra et al., 2022; Saul et al., 2024) or acute lung injury (Blázquez‐Prieto et al., 2021), tended to activate p21‐related pathways of cellular senescence. *CDKN1A*+ non‐replicating cells were more congruent with SenSkin™ and SenMayo than *CDKN2A*+ non‐replicating cells, suggesting that *CDKN1A*+ non‐replicating cells in vivo produced a more pronounced SASP, as previously suggested (Sturmlechner et al., 2021), and other senescence‐associated genes identified in human skin. Differences observed between *CDKN1A*+ and *CDKN2A*+ non‐replicating cells could suggest that senescence induction in the skin predominantly originates from oxidative stress, telomere attrition, or oncogenic stress (Kumari & Jat, 2021), and it could also reflect a tendency for cells in the skin, especially the dermis, to express p21 rather than p16 (McClusky et al., 2020). Finally, supporting the unitary theory of fundamental aging processes, cellular senescence in dermal fibroblasts, particularly in late phases, was associated with loss of proteostasis.

This study also connects senescent cells to clinical signs of skin aging. In the epidermis, senescent melanocytes were associated with increased melanin synthesis, consistent with the role of melanin in protection from UV radiation and oxidative stress (Hughes & Bishop, 2022). Elevated melanin synthesis could be associated with age‐related dyschromia or dyspigmentation, particularly in the context of photoaging. In the dermis, senescent reticular dermal fibroblasts were associated with reduced collagen and elastic fiber synthesis, which is also consistent with age‐related changes such as soft tissue volume loss and wrinkling. This finding is also consistent with the senescence‐dependent restriction of fibrosis observed in acute cutaneous wounds (Jun & Lau, 2010). However, senescent fibroblasts have been implicated in lung and heart fibrosis, indicating a location‐ or lineage‐dependent effect of cellular senescence on fibroblasts (Schafer et al., 2017; Suda et al., 2023). These results suggest that senescent cell elimination or modulation may reduce clinical signs of skin aging. Future in vitro and in vivo studies are needed to confirm the changes to melanin and ECM synthesis in melanocytes and fibroblast subpopulations. Moreover, additional studies are required to corroborate findings in senescent cell lineage trajectories due to limitations in current trajectory inference techniques.

In this study, the spatial transcriptomic analyses support the hypothesis that senescent cells display a tendency to cluster. Including spatial orientation and clustering behaviors of senescent cells could guide accurate in vitro models of aging tissues. Moreover, future studies could be directed at understanding how senescent cells cluster, whether paracrine signaling causes neighboring cells to become senescent, or if senescent cells migrate towards each other. Analyses could also be performed to explore the heterogeneity and organization of senescent cell clusters. Methods we developed to assess spatial clustering or dispersion of cells could be applied generally to evaluate the tendency for any subpopulation of cells to cluster or disperse, given Visium sequencing data, which is based on discrete hexagonal grids. These findings portend a novel architectural blueprint of the epidermis and dermis based on hallmarks of aging for human skin.

## 4 MATERIALS AND METHODS

### 4.1 Experimental design

To characterize senescent cells in skin, scRNA‐seq datasets of normal human full‐thickness skin were obtained. Datasets that sequenced skin from locations on the face, which is more sun‐exposed, and the body, which is less sun‐exposed, were compared to evaluate differences due to sun exposure, a known cause of skin aging. Specifically, scRNA‐seq data were from Ganier, et al., from the Human Cell Atlas (Ganier et al., 2024), a study by Solé‐Boldo et al. 2020, and a study by Tabib et al. 2018. Altogether, they included scRNA‐seq of 15 normal human full‐thickness skin samples collected from sun‐exposed skin in the face (13 male, 2 female; average age: 77 years) and 11 from non‐sun‐exposed areas in the body (8 male, 3 female; average age: 49 years). For scRNA‐seq, samples were immediately dissociated into single‐cell suspension or stored in MACS Tissue Storage Solution no longer than overnight prior to dissociation; more details on sample processing can be found in the primary articles (Ganier et al., 2024; Solé‐Boldo et al., 2020; Tabib et al., 2018).

Because of the high‐dimensional scRNA‐seq data, senescent cells could be identified by combining canonical cellular senescence markers, *CDKN1A* and *CDKN2A*, cell cycle analysis, and other senescence‐associated genes, including SASP. We tested our method for identifying senescent cells by observing senescent cell burdens with sun exposure and chronological age. Further, we explored and characterized senescent cell.

To observe the spatial distribution of senescent cells in skin, Visium spatial transcriptomic sequencing data were obtained from Ganier, et al. and included 13 samples of sun‐exposed skin from the face (11 male, 2 female; average age: 61 years) and 9 samples of non‐sun‐exposed skin from the body (8 male, 1 female; average age: 54 years) (Ganier et al., 2024). Samples were fresh frozen and OCT‐embedded, then cryosectioned at 10 μm onto Visium slides (10X Genomics) and processed according to the manufacturer's protocol (Ganier et al., 2024). Because senescent cells have been described to cluster in other tissues, we statistically tested their tendency to cluster. We observed whether the tendency for senescent cells to cluster changed with sun exposure and chronological age. Because Visium spatial transcriptomic sequencing data had a lower gene limit, we used high *CDKN1A* expression to identify senescent cells.

### 4.2 ScRNA‐seq preprocessing and analysis

All scRNA‐seq analysis was performed in R v4.3.2. ScRNA‐seq datasets that had first been preprocessed using CellRanger, v6.1.1 (10X Genomics). Next, Seurat v5.0.1 was used to further preprocess and analyze the data (Hao et al., 2021). As previously described, datasets were combined using Seurat's merge() function with the criteria “min.cells = 3” and “min.features = 200” (Ganier et al., 2024). To exclude potential cell doublets, cells with greater than 5000 expressed genes were removed and to exclude apoptotic cells, cells with greater than 5% mitochondrial reads were removed.

Datasets were then integrated using Seurat's reference‐based integration protocols with correction of batch effects from differences among samples. First, data were log‐normalized by unique molecular identifier counts and identification of the 2000 most variable genes in each sample. Then, integration anchors were identified using the FindIntegrationAnchors() function with default parameters and 20 canonical correlation analysis dimensions. The anchors were used to integrate the data using IntegrateData() function. The integrated data, which include the 2000 most variable genes, were used for cell clustering and visualization, while the full dataset was used for gene and gene set analysis to maximize genes available for analysis.

Data were scaled using the ScaleData() function, log‐normalized using the NormalizeData() function, and principal component analysis (PCA) was performed using the RunPCA() function. Unsupervised clustering of cells was performed using the FindNeighbors() and FindClusters() functions, using the first 20 PCA dimensions and a resolution of 0.65. Dimension reduction was performed using the RunUMAP() function with default parameters and 20 PCA dimensions for visualization. Differential gene expression for different clusters or subsets of cells was evaluated using the FindMarkers() and FindAllMarkers() functions, which use Wilcoxon rank‐sum tests with a fold change cutoff of 0.25 (natural log scale) and a Bonferroni‐adjusted p‐value cutoff of 0.05. Cell types were described based on independent established markers of human skin cell types.

A cell's positivity for a marker, that is, *CDKN1A* or *CDKN2A*, was defined by a log‐normalized expression threshold greater than 0.5 after observing the distribution of *CDKN1A* and *CDKN2A* expression. For cell cycle analysis, each cell was classified as being in either G2M (G2 or mitosis), S, or G1 (including G0) phase using the CellCycleScoring() function. Standard cell cycle markers for S phase and G2M phase from Seurat were used to calculate scores for classification (Tirosh et al., 2016).

R packages ggplot2 v3.4.4, ggrepel v0.9.4, and RColorBrewer v1.1.3 were used to improve clarity of visualizations. R package ggpubr v0.6.0 was used for violin plot statistics. Specifically, the function stat_compare_means() was used to perform Wilcoxon signed‐rank tests between two groups or Kruskal–Wallis tests between three or more groups.

### 4.3 Pathway enrichment analysis

A web‐based gene set analysis toolkit (WebGestalt; www.webgestalt.org/) was used for pathway enrichment analysis to identify gene sets overrepresented by upregulated SenSkin™ genes. Inputs were represented by lists of gene symbols, and they were analyzed for overrepresentation against the *Homo sapiens* genome reference set. Pathways were obtained from the non‐redundant biological process functional database from Gene Ontology (Elizarraras et al., 2024).

### 4.4 Single‐cell gene set scoring

Composite scores to evaluate overall enrichment of expression in sets of genes for each cell were calculated using a normalized sum of *z*‐scores. All gene expression values were log‐normalized. Then, expression values of each gene in the gene set were standardized to obtain *z*‐scores. For each cell, the sum of *z*‐scores for all genes in the gene set was obtained and normalized by the number of genes in the gene set.

The precise mathematical expression of composite scores is given for each cell i by equation (1):

(1) $$\text{Composite score}=\sum_{j=1}^{m_{i}}\frac{z_{\mathrm{ij}}}{m_{i}}-\sum_{l=1}^{k_{i}}\frac{z_{\mathrm{ik}}}{k_{i}}$$

where there exists n cells, $m_{i}$ genes that are expected to be upregulated, and $k_{i}$ genes that are expected to be downregulated. Gene value standardizations for each cell are given by equation (2):

(2) $$\begin{array}{c} z_{\mathrm{ij}}=\frac{x_{\mathrm{ij}}-\bar{x_{j}}}{\sigma_{x_{j}}}\; \end{array}$$

where $x_{\mathrm{ij}}$ is the log‐normalized gene expression value for cell i and gene j, the mean is given by $\bar{x_{j}}=\sum_{i=1}^{n}\frac{x_{\mathrm{ij}}}{n}$, and the standard deviation is given by $\sigma_{x_{j}}=\sqrt{\frac{\sum_{i=1}^{n}{\left(x_{i}-\bar{x}\right)}^{2}}{n}}$.

### 4.5 Trajectory inference (pseudotime) analysis

Trajectory inference analysis was performed using Monocle 3 v1.2.7 after transforming the Seurat dataset to a Monocle 3 cds dataset using SeuratWrappers v0.3.2. The function preprocess_cds() was used to normalize the data, reduce_dimension() to perform UMAP dimension reduction to 25 dimensions, and cluster_cells() to cluster the cells with the default resolution of 1 × 10^−5^. Root nodes of pseudotime genes were selected by identifying cell clusters with lowest *CDKN1A* expression. Top markers in each cell cluster were identified using the fit_models() function for regression analysis, followed by the top_markers() function. Genes that changed as a function of pseudotime were identified using the graph_test() function.

### 4.6 Spatial transcriptomic (Visium) data analysis

Space Ranger v1.3.0 (10X Genomics) outputs were imported into Seurat. As previously described, spots with greater than 30% mitochondrial genes and less than 200 genes, or spots containing necrotic or damaged areas were removed. Normalization was performed using the SCTranform() function. PCA and dimension reduction were performed using the same method as for scRNA‐seq. Data were visualized as overlaid on hematoxylin and eosin images using the SpatialFeaturePlot() function.

Spatial clustering of *CDKN1A* or *CDKN2A*‐expressing or highly expressing spots was calculated using point distances with the raster v3.6.26 package. Positive spots were defined to be in clusters if they had a minimum point distance of 1, which indicated being adjacent to another positive spot. For each sample, the number of positive spots and the spatial distribution of all spots were used to simulate the number of spots in clusters given random chance. Ten thousand simulations were used to produce a null distribution for numbers of spots in clusters, because this yielded an approximately normal distribution for most cases as evaluated by QQ plots. The number of spots in clusters in each sample was compared to the simulated distribution, yielding the percentile, and a one‐sided Wilcoxon signed‐rank test was performed, yielding a *p*‐value. Full code for the spatial clustering analysis can be found at https://github.com/grace‐y/spatialcluster.

### 4.7 Statistical analysis

Differential gene expression for different clusters or subsets of cells was evaluated using the FindMarkers() and FindAllMarkers() functions, which use Wilcoxon rank‐sum tests with a fold change cutoff of 0.25 (natural log scale) and a Bonferroni‐adjusted *p*‐value cutoff of 0.05. Violin plot statistics were performed using the function stat_compare_means(), which performed Wilcoxon signed‐rank tests between two groups or Kruskal–Wallis tests between three or more groups. Congruence statistics were performed using a binomial distribution. The proportion of congruent genes in one group was compared to the proportion of congruent genes in the other group using a one‐sided exact binomial test. Other specific statistical analyses were described in the corresponding method sections.

## AUTHOR CONTRIBUTIONS

Conceptualization: G.T.Y., C.G., T.T., J.L.K., M.D.L., and S.P.W. Methodology: G.T.Y., C.G., D.B.A., T.T., J.L.K., M.D.L., and S.P.W. Investigation: G.T.Y., C.G., M.D.L., and S.P.W. Visualization: G.T.Y. and C.G. Supervision: D.B.A., T.T., J.L.M., M.D.L., and S.P.W. Writing – original draft: G.T.Y. Writing – reviewing and editing: G.T.Y., C.G., D.B.A., T.T., S.K., J.L.K., M.D.L., and SPW.

## FUNDING INFORMATION

This work was supported by the National Institutes of Health [grant numbers T32 GM065841, R03AG082919–01, R37AG013825, R33AG061456, P01AG062413, R01 AG076515, U54AG079754, P30AG050886, U24AG056053]. It was also supported by the Wellcome Trust [grant numbers 211276/E/18/Z, 096540/Z/11/Z]. In addition, the work was supported by Hevolution Foundation, the Connor Fund, the Noaber Foundation, Pfizer, Inc, the Gordon and Betty Moore Foundation, the National Cattlemen's Beef Association, Weight Watchers, the National Pork Board, and the Novo Nordisk Foundation.

## CONFLICT OF INTEREST STATEMENT

J.L.K. and T.T. have financial interest related to this research, including patents, and pending patents covering senolytic drugs and their uses that are held by Mayo Clinic. This research has been reviewed by the Mayo Clinic Conflict of Interest Review Board and was conducted in compliance with Mayo Clinic and Cedars‐Sinai conflict of interest policies. M.D.L. is a co‐founder of Fibrodyne and has two patents related to skin fibroblasts. In the last twenty‐four months, D.B.A. has received personal payments or promises for same from: Novo Nordisk Foundation; and Zero Longevity Science (as stock options). D.B.A.'s institution, Indiana University, and the Indiana University Foundation have received funds or donations to support his research or educational activities from: Eli Lilly and Company; Pfizer, Inc.; and numerous other for‐profit and non‐profit organizations to support the work of the School of Public Health and the university more broadly.

## PERMISSION STATEMENT

No material enclosed was reproduced. Reproduction, distribution or transmission is prohibited, except as otherwise permitted by law.

## Supporting information


Data S1.

## Data Availability

No primary data generation was performed in this study. All code is available in the main text or the supplementary materials.
