# Supplementary material for: Mapping epidermal and dermal cellular senescence in human skin aging
Source: Aging Cell. 2024 Oct 6;24(1):e14358. doi: 10.1111/acel.14358 (PMC11709101; doi:10.1111/acel.14358)
Supplement: Supplementary file 1 — Data S1. [file ACEL-24-e14358-s001.docx]

**Supporting Information**

**Mapping epidermal and dermal cellular senescence in human skin aging**

Grace T. Yu, BSc, Clarisse Ganier, PhD, David B. Allison, PhD, Tamara Tchkonia, PhD, Sundeep Khosla, MD, James L. Kirkland, MD, PhD, Magnus D. Lynch, MRCS, DPhil, Saranya P. Wyles, MD, PhD*


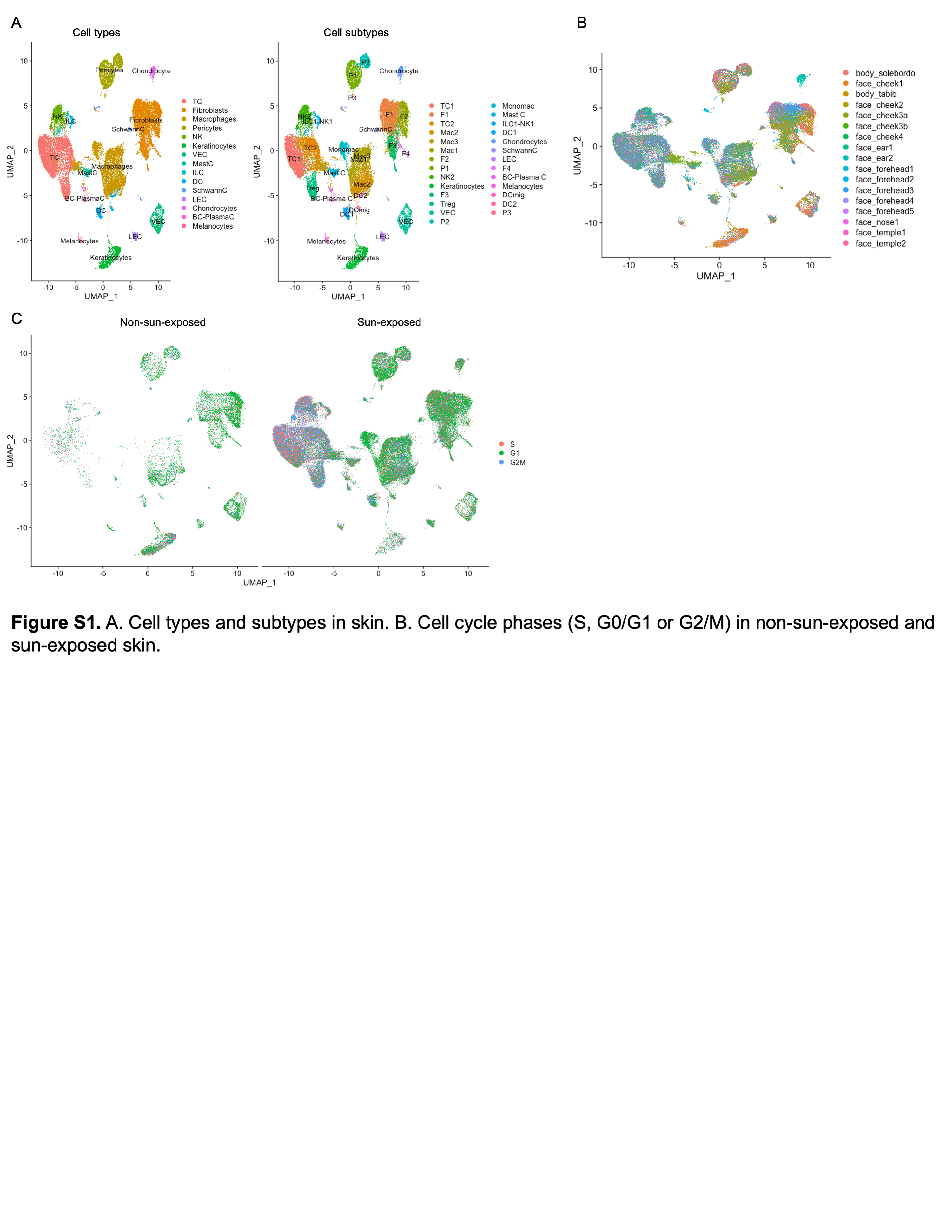


Fig. S1. (A), Cell types and subtypes in skin. (B), UMAP clustering by donor/batches. (C), Cell cycle phases (S, G0/G1 or G2/M) in non-sun-exposed and sun-exposed skin.


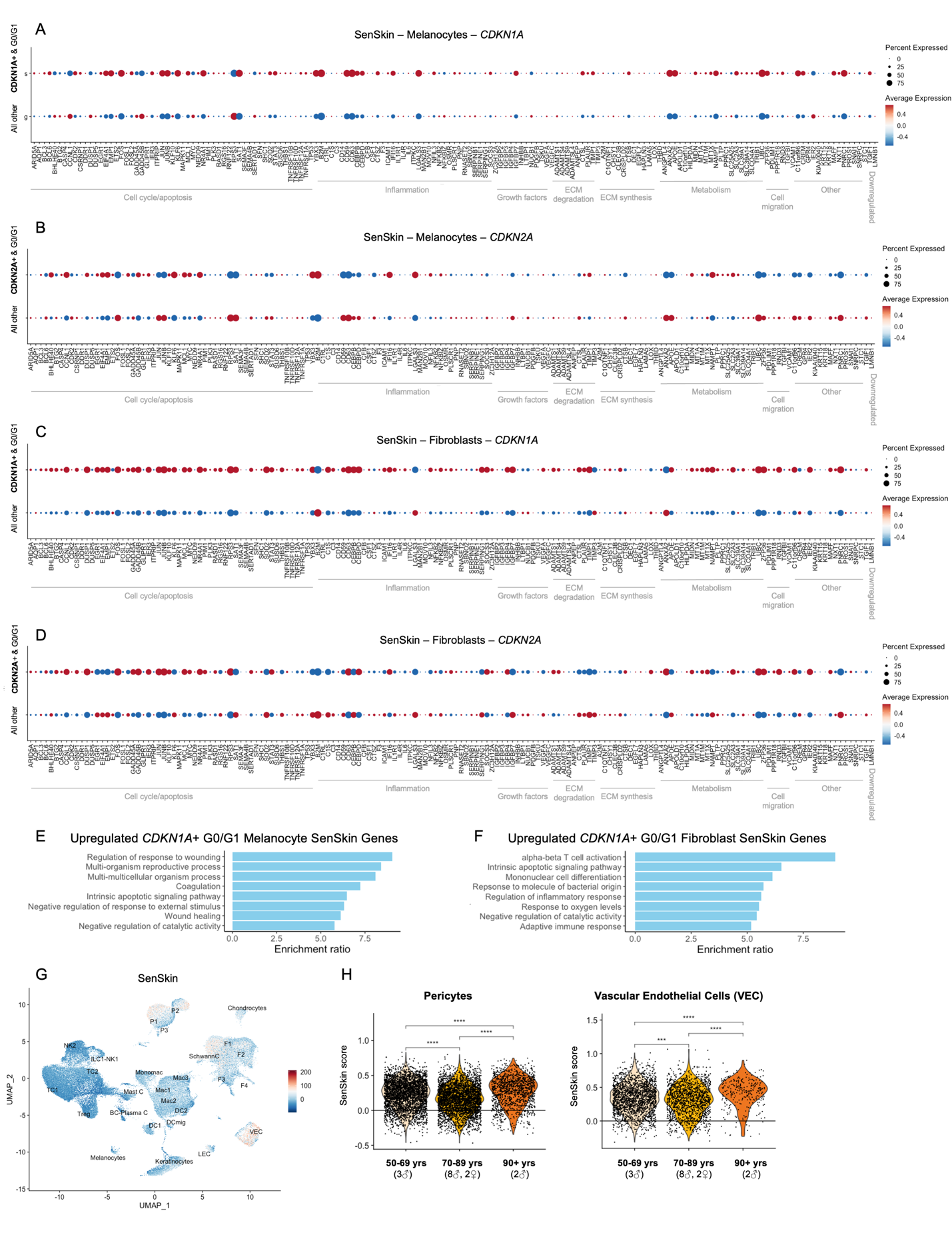


Fig. S2. SenSkin, grouped approximately by gene role, in non-replicating (A), *CDKN1A*+ melanocytes (106/165 congruent genes) or (B), *CDKN2A*+ melanocytes (65/165 congruent genes) *vs*. other melanocytes. SenSkin was more congruent with *CDKN1A*+ non-replicating melanocytes than *CDKN2A*+ non-replicating melanocytes (*p* = 1.0x10^-10^). Red dots represent gene upregulation, and blue dots represent gene downregulation. Dot size represents percent of cells expressing the gene. SenSkin in non-replicating (C), *CDKN1A*+ fibroblasts (120/165 congruent genes) or (D), *CDKN2A*+ fibroblasts (78/165) *vs*. other fibroblasts. SenSkin was more congruent with *CDKN1A*+ non-replicating fibroblasts than *CDKN2A*+ non-replicating fibroblasts (*p* = 2.8x10^-11^). Top Gene Ontology Biological Process gene sets overrepresented by SenSkin genes upregulated by *CDKN1A*+ non-replicating (E) melanocytes and (F) fibroblasts. All gene sets listed have FDR < 0.05. (G), SenSkin composite score across cell types in skin. (H), SenSkin *vs*. chronological age in cell types with significant differences (melanocytes, keratinocytes, and fibroblasts are presented in subsequent figures). ns: p > 0.05; *: p <= 0.05; **: p <= 0.01; ***: p <= 0.001; ****: p <= 0.0001.


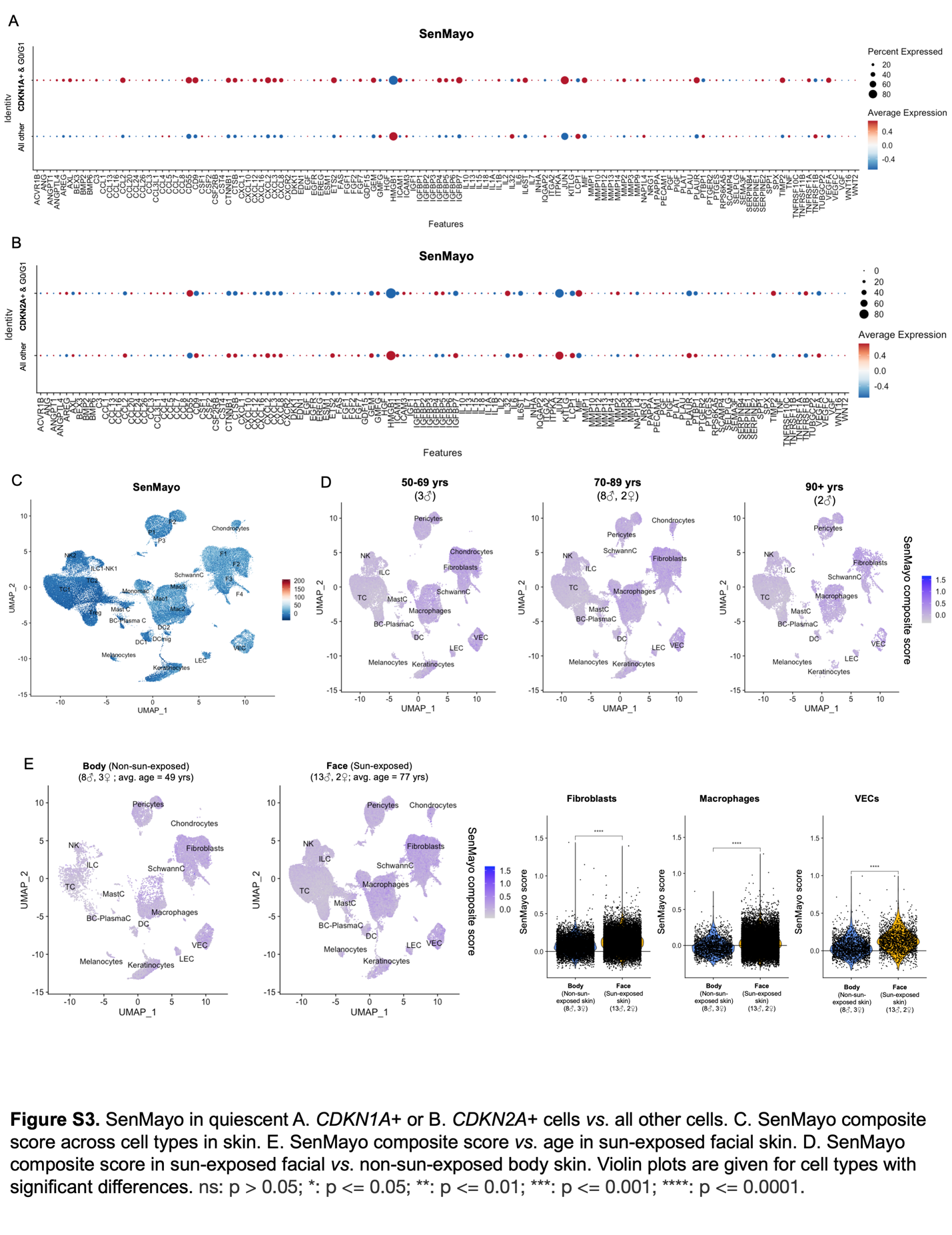


Fig. S3. SenMayo in non-replicating (A), *CDKN1A*+ cells (93/125 congruent genes) or (B), *CDKN2A*+ cells (50/125 congruent genes) *vs*. all other cells. SenMayo was more congruent with *CDKN1A*+ non-replicating cells than *CDKN2A*+ non-replicating cells (*p* = 6.2x10^-15^). Red dots represent gene upregulation, and blue dots represent gene downregulation. Dot size represents percent of cells expressing the gene. (C), SenMayo composite score across cell types in skin. (D), SenMayo composite score in sun-exposed facial *vs*. non-sun-exposed body skin. (E), SenMayo composite score *vs*. age in sun-exposed facial skin. Violin plots are given for cell types with significant differences. ns: p > 0.05; *: p <= 0.05; **: p <= 0.01; ***: p <= 0.001; ****: p <= 0.0001.


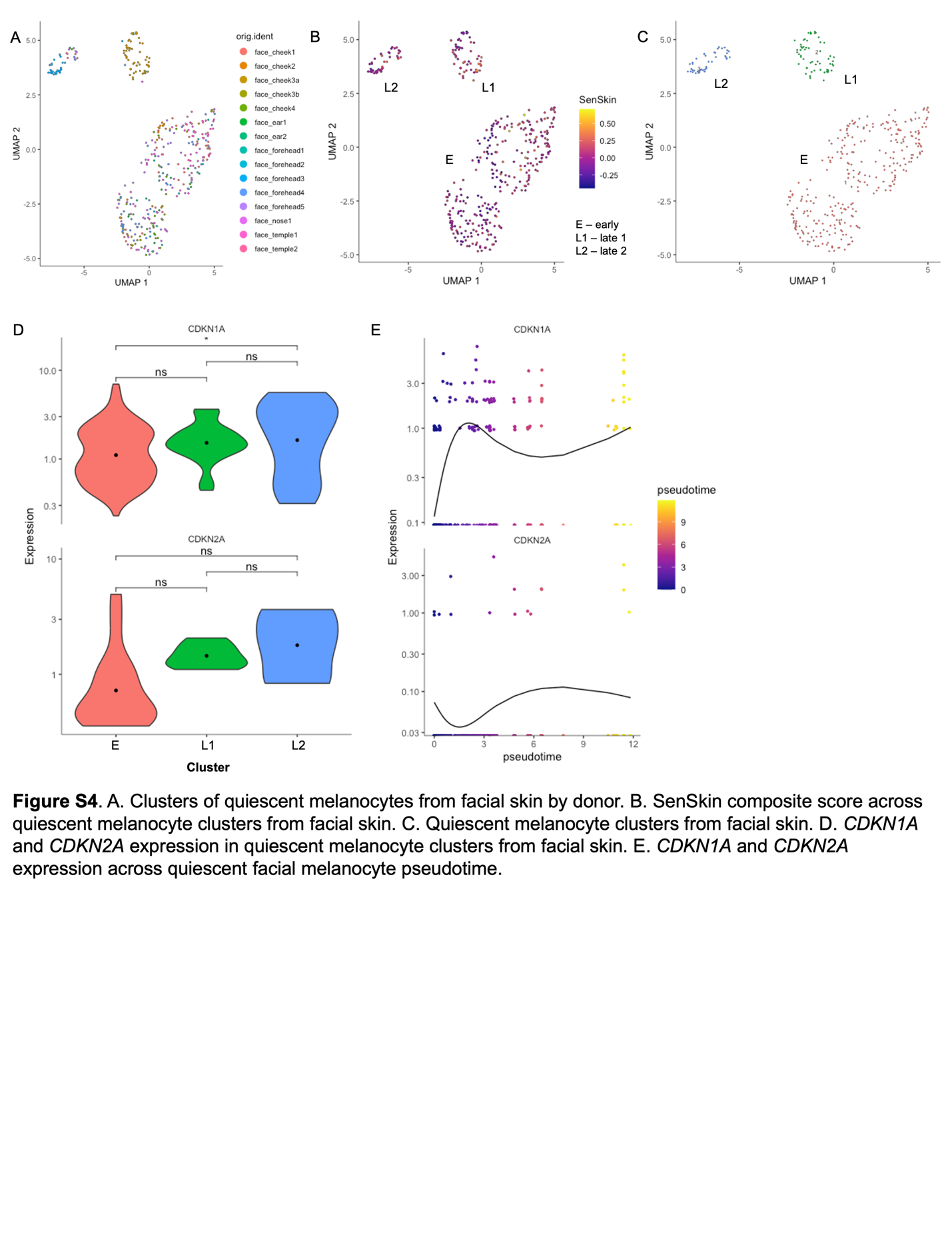


Fig. S4. (A), Clusters of non-replicating melanocytes from facial skin by donor. (B), SenSkin composite score across non-replicating melanocyte clusters from facial skin. (C), Non-replicating melanocyte clusters from facial skin. (D), *CDKN1A* and *CDKN2A* expression in non-replicating melanocyte clusters from facial skin. (E), *CDKN1A* and *CDKN2A* expression across non-replicating facial melanocyte pseudotime.


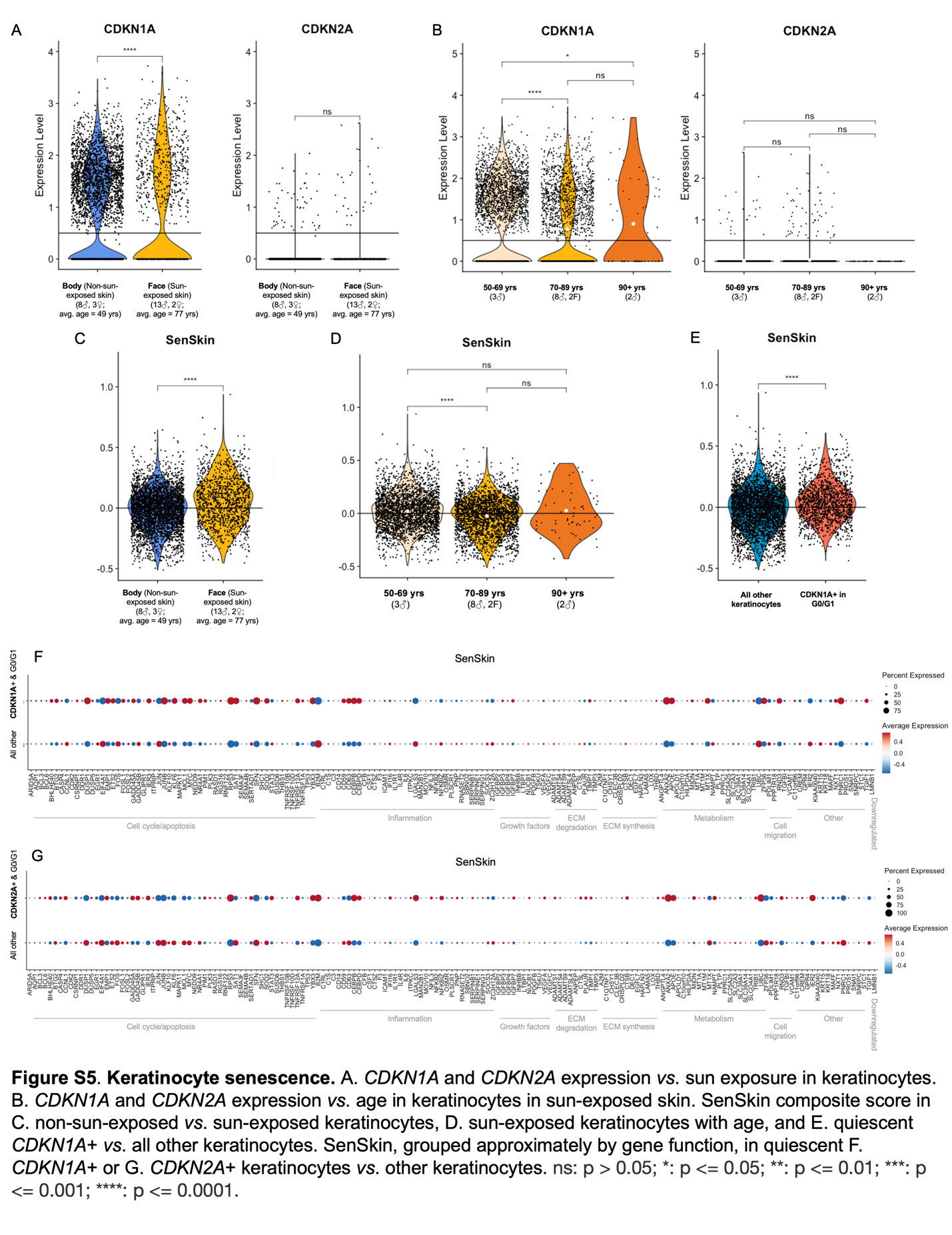
Fig. S5. Keratinocyte senescence. (A), *CDKN1A* and *CDKN2A* expression *vs*. sun exposure in keratinocytes. (B), *CDKN1A* and *CDKN2A* expression *vs*. age in keratinocytes in sun-exposed skin. SenSkin composite score in (C), non-sun-exposed *vs*. sun-exposed keratinocytes, (D), sun-exposed keratinocytes with age, and (E), non-replicating *CDKN1A*+ *vs*. all other keratinocytes. SenSkin, grouped approximately by gene function, in non-replicating (F), *CDKN1A*+ or (G), *CDKN2A*+ keratinocytes *vs*. other keratinocytes. Red dots represent gene upregulation, and blue dots represent gene downregulation. Dot size represents percent of cells expressing the gene. ns: p > 0.05; *: p <= 0.05; **: p <= 0.01; ***: p <= 0.001; ****: p <= 0.0001.


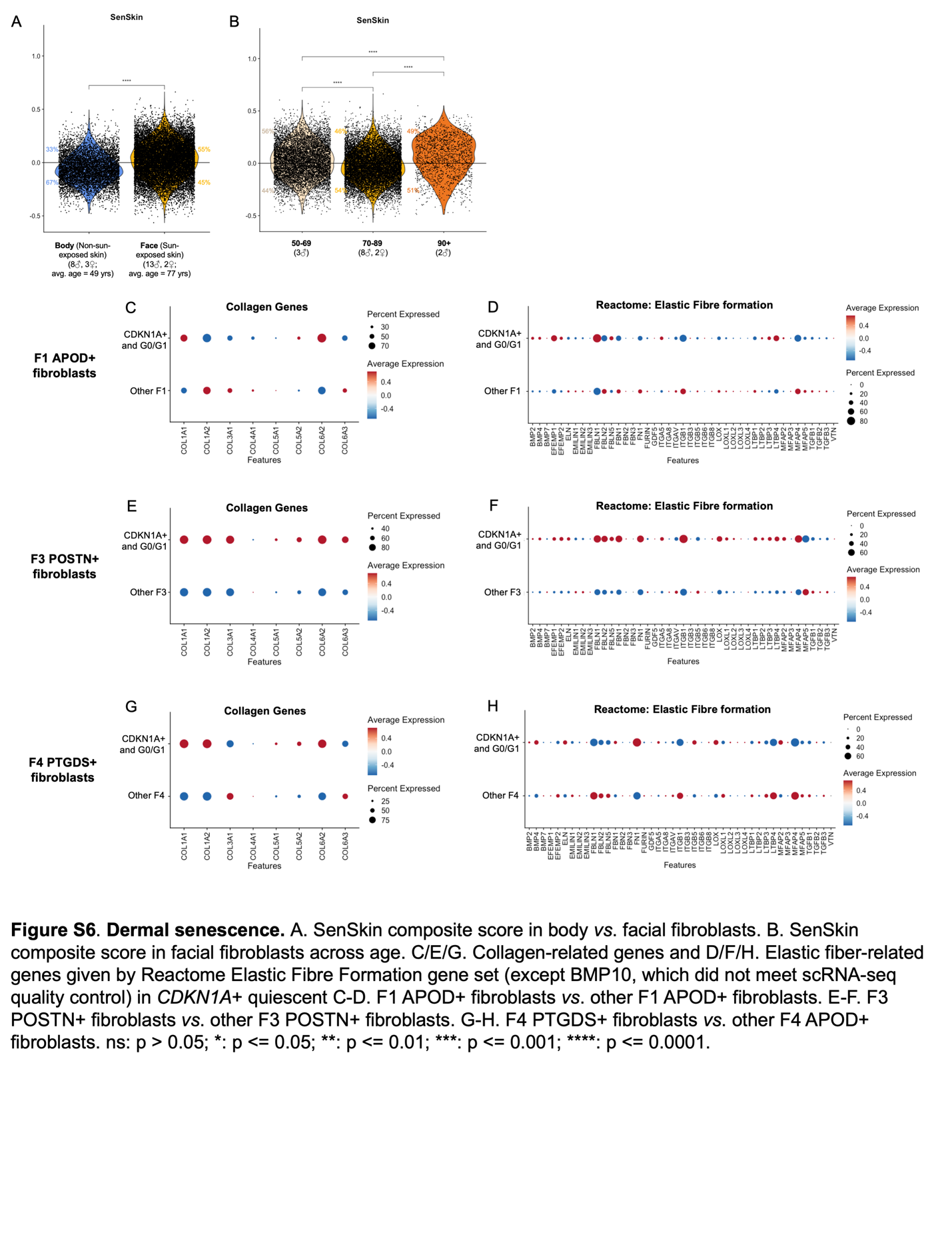


Fig. S6. Dermal senescence. (A), SenSkin composite score in body *vs*. facial fibroblasts. (B), SenSkin composite score in facial fibroblasts across age. (C/E/G), Collagen-related genes and (D/F/H), Elastic fiber-related genes given by Reactome Elastic Fibre Formation gene set (except BMP10, which did not meet scRNA-seq quality control) in *CDKN1A*+ non-replicating (C-D), F1 APOD+ fibroblasts *vs*. other F1 APOD+ fibroblasts. (E-F), F3 POSTN+ fibroblasts *vs*. other F3 POSTN+ fibroblasts. (G-H), F4 PTGDS+ fibroblasts *vs*. other F4 APOD+ fibroblasts. Red dots represent gene upregulation, and blue dots represent gene downregulation. Dot size represents percent of cells expressing the gene. ns: p > 0.05; *: p <= 0.05; **: p <= 0.01; ***: p <= 0.001; ****: p <= 0.0001.


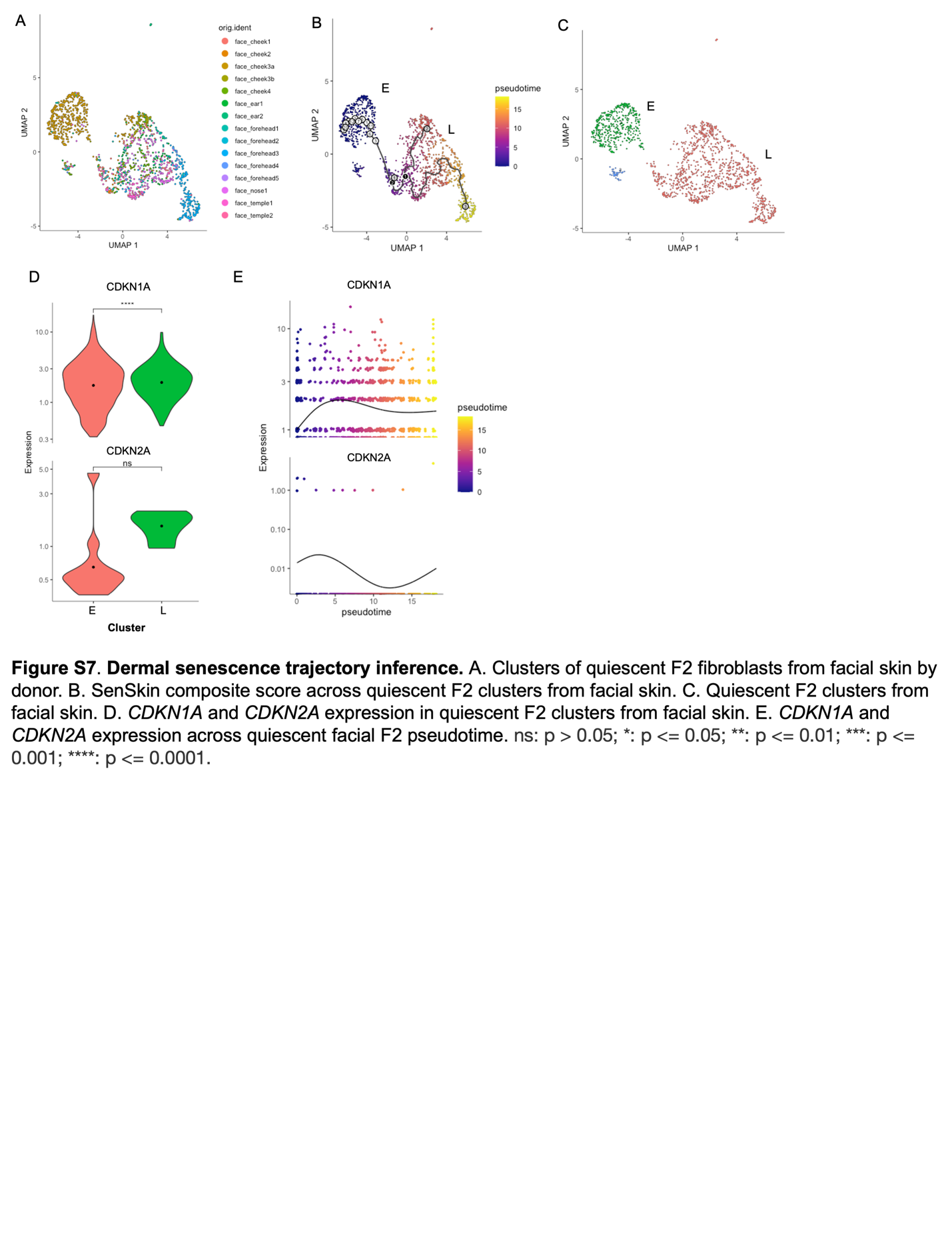
Fig. S7. Dermal senescence trajectory inference. (A), Clusters of non-replicating F2 fibroblasts from facial skin by donor. (B), SenSkin composite score across non-replicating F2 clusters from facial skin. (C), Non-replicating F2 clusters from facial skin. (D), *CDKN1A* and *CDKN2A* expression in non-replicating F2 clusters from facial skin. (E), *CDKN1A* and *CDKN2A* expression across non-replicating facial F2 pseudotime. ns: p > 0.05; *: p <= 0.05; **: p <= 0.01; ***: p <= 0.001; ****: p <= 0.0001.


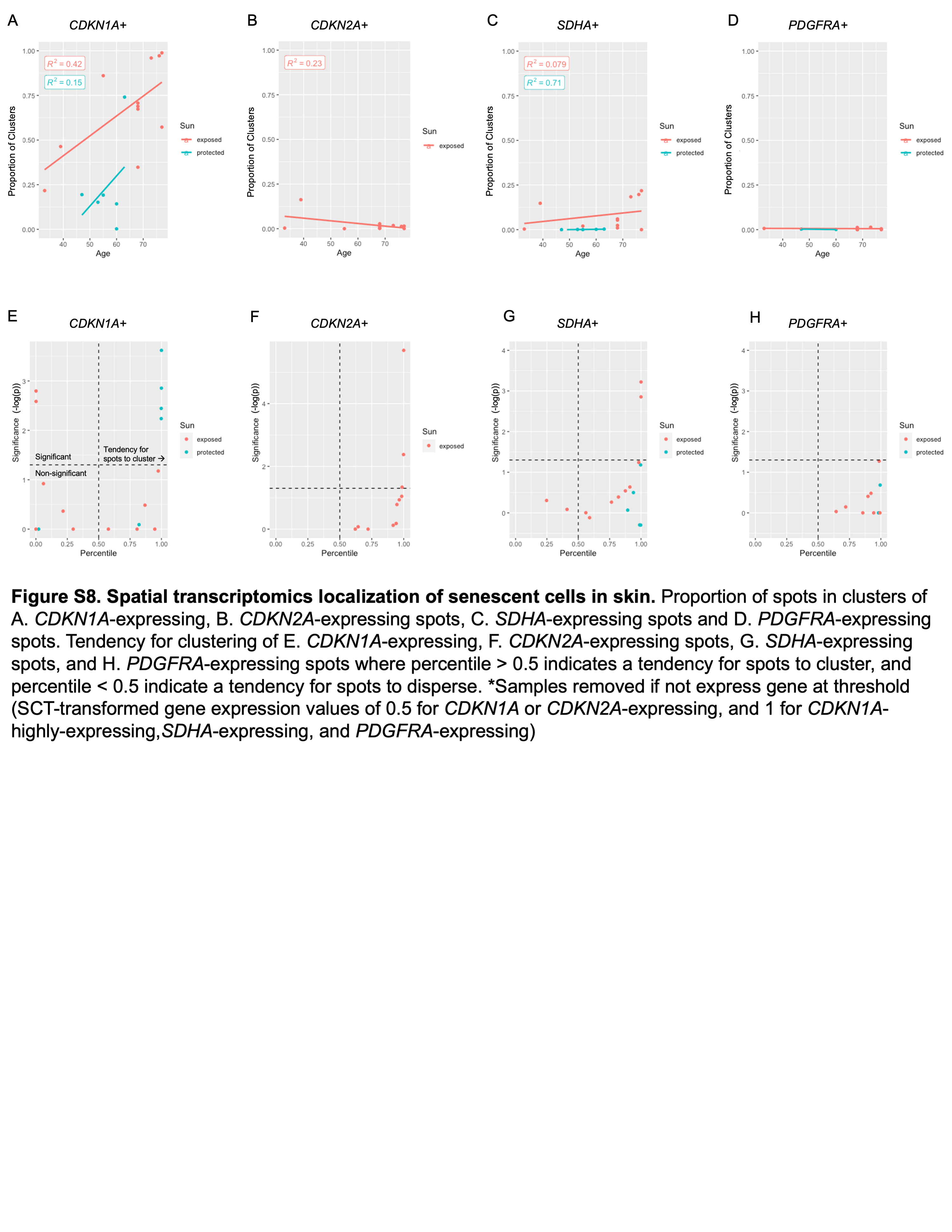


Fig. S8. Spatial transcriptomics localization of senescent cells in skin. Proportion of spots in clusters of (A), *CDKN1A*-expressing, (B), *CDKN2A*-expressing spots, (C), *SDHA*-expressing spots and (D), *PDGFRA*-expressing spots. Tendency for clustering of (E), *CDKN1A*-expressing, (F), *CDKN2A*-expressing spots, (G), *SDHA*-expressing spots, and (H), *PDGFRA*-expressing spots where percentile > 0.5 indicates a tendency for spots to cluster, and percentile < 0.5 indicate a tendency for spots to disperse. *Samples removed if not express gene at threshold (SCT-transformed gene expression values of 0.5 for *CDKN1A* or *CDKN2A*-expressing, and 1 for *CDKN1A*-highly-expressing, *SDHA*-expressing, and *PDGFRA*-expressing).

| Genes |
| --- |
| ARID5A |
| AQP1 |
| BCL3 |
| BCL6 |
| BHLHE40 |
| BTG2 |
| CASP4 |
| CCNL1 |
| CDK2 |
| CSRNP1 |
| DDR1 |
| DUSP1 |
| DUSP5 |
| EGR1 |
| EIF4A1 |
| EMP1 |
| ETS2 |
| FOS |
| FOSL1 |
| FOSL2 |
| GADD45A |
| GADD45B |
| GLIPR1 |
| IER3 |
| ITPRIP |
| JUN |
| JUNB |
| KLF10 |
| KLF6 |
| MAPK11 |
| MCL1 |
| MYC |
| NEDD9 |
| NR4A1 |
| PIM1 |
| PLK3 |
| RASD1 |
| RGS16 |
| RNF122 |
| RPS3 |
| SAT1 |
| SEMA3F |
| SEMA4B |
| SERTAD1 |
| SFN |
| SHC1 |
| SOD2 |
| STAT3 |
| SUSD6 |
| THBS1 |
| TNFRSF10B |
| TNFRSF10D |
| TNFRSF12A |
| TNFRSF1A |
| TP53 |
| YBX3 |
| B2M |
| C1RL |
| C1S |
| C3 |
| CD14 |
| CD59 |
| CD63 |
| CEBPB |
| CEBPD |
| CFB |
| CSF1 |
| CTSZ |
| F3 |
| ICAM1 |
| IFI16 |
| IL1R1 |
| IL4R |
| IL6 |
| ITPKC |
| LGALS3 |
| MAN2B1 |
| MOV10 |
| NFIL3 |
| NFKB2 |
| NFKBIZ |
| OSMR |
| PLSCR1 |
| PNP |
| RNASET2 |
| SBNO2 |
| SERPINB1 |
| SERPINE1 |
| SERPING1 |
| SOCS3 |
| ZC3H12A |
| IGFBP2 |
| IGFBP3 |
| IGFBP4 |
| IGFBP7 |
| INHBB |
| LTBP1 |
| NUCB1 |
| PDGFB |
| TSKU |
| VEGFA |
| VEGFC |
| ADAMTS1 |
| ADAMTS4 |
| ADAMTS9 |
| ADAMTSL4 |
| ANPEP |
| CTSL |
| PLAUR |
| TIMP1 |
| TIMP3 |
| A2M |
| C1QTNF1 |
| CHSY1 |
| CLEC3B |
| CRISPLD2 |
| CTSB |
| DEC1 |
| EGFL7 |
| HAPLN3 |
| LAMA5 |
| LOX |
| THBD |
| ANGPTL4 |
| ANXA2 |
| APOE |
| APOLD1 |
| C10orf10 |
| HILPDA |
| MIDN |
| MT1A |
| MT1M |
| MT1X |
| NAMPT |
| PLTP |
| PPRC1 |
| SLC25A25 |
| SLC2A3 |
| SLC39A1 |
| SLC39A14 |
| SLCO4A1 |
| TRIB1 |
| UBC |
| ZFP36 |
| PDLIM1 |
| PPP1R18 |
| RND3 |
| TGFBI |
| VCAM1 |
| C11orf96 |
| CREM |
| GPR4 |
| IER2 |
| KIAA0040 |
| KRT15 |
| KRT18 |
| MAFF |
| NXT1 |
| PNRC1 |
| PROS1 |
| SNAI1 |
| SNRPC |
| STC1 |
| TGIF1 |
| LMNB1 |

Table S1. SenSkin genes.
